# Psychometric properties of a Silhouette Rating Scale assessing current and ideal body size and body dissatisfaction in adults

**DOI:** 10.1007/s40519-021-01258-6

**Published:** 2021-07-08

**Authors:** Caterina Lombardo, Silvia Cerolini, Rita Maria Esposito, Fabio Lucidi

**Affiliations:** 1 Department of Psychology, Sapienza University of Rome, via dei Marsi 78, 00185 Rome, Italy; 2 Department of Development and Socialization Processes Psychology, Sapienza University of Rome, Rome, Italy

**Keywords:** Body size, Body image, Body dissatisfaction, Analogic scale, Assessment

## Abstract

**Purpose:**

The study aims at validating a new pictorial tool, the Silhouette Rating Scale (SRS). It consists of a series of nine female or male silhouettes. It was created to assess current and ideal body size evaluation, and body dissatisfaction. Our aims were to test the concurrent, convergent and discriminant validity of the scale, evaluating possible gender differences.

**Method:**

A first sample of 754 young adults (age *M* = 26.10 ± 8.50, males *N* = 218) and a second sample of 210 young adults (age *M* = 21.19 ± 3.22, males = 43) completed the SRS, and other self-report measures assessing body size evaluation, disordered eating, body satisfaction, depression, emotion regulation and insomnia.

**Results:**

Statistical analyses performed on the first sample largely support the concurrent validity of the scale. Results obtained from the second sample confirm its convergent validity, showing strong correlations with the Contour Drawing Rating Scale. In addition, the correlations performed between the three responses of the SRS and other measures of eating disorders, depression, insomnia and emotion regulation indicated a good discriminant validity, though some of the variables measured seem to be significantly correlated.

**Conclusions:**

The SRS is a reliable and valid tool for assessing current body size, body ideal and body dissatisfaction as compared to other widely used scales. It guarantees the universality of use thanks to the absence of details related to ethnicity or culture and at the same time, maintaining a right level of realism. Future studies will evaluate test–retest validity and its potential within clinical populations.

**Leve of evidence:**

V, descriptive cross sectional study

**Supplementary Information:**

The online version contains supplementary material available at 10.1007/s40519-021-01258-6.

## Introduction

Body image attitudes are assumed to play a central role in organizing thoughts, behaviors, feelings, and evaluations related to one’s body [1]. Negative body image attitudes may manifest by strong importance of as well as a negative appreciation of one’s shape and weight, i.e. body dissatisfaction. Body dissatisfaction is a form of distress related to one's own body that is associated with a perceptually distorted image of one's shape or body size and a definite feeling of discomfort. Those feelings can vary from the slight embarrassment of the appearance of some parts of the body to the actual avoidance of the exposure of one's body (for example, not going to the pool or changing in front of others in the changing rooms of a gym) even at yourself (for example, avoid looking in the mirror).

The study of the body image has grown in recent years, parallel to an increase in the number of new or revised measures for its evaluation [for a review, see 2]. Self-report measures of body image are good instruments for capturing feelings of discomfort, attitudes towards one’s body and behaviors like avoidance of facing one’s body. However, they are susceptible to the influence of social desirability and self-deception. Moreover, they need language and cultural adaptation for being used in different countries [3].

Some tools available for the evaluation of body dissatisfaction are described as pictorial, meaning they involve the use of figures or images. Among the most used analog instruments, there are certainly the Figure Rating Scale (FRS) [4]; and the Contour Drawing Rating Scale (CDRS) [5] tools of smooth and short administration. In both scales, it is asked to choose from nine silhouettes depicting images of bodies of various sizes, and gender and age appropriate. Respondents are asked to choose the image that better represents their actual size. Then, they are asked to choose which image represents their ideal body size. The first is considered a measure of perception of one's body (current body image); the second is understood as an adherence to the ideals of beauty socially shared in the culture to which the respondent belongs (ideal body image). The discrepancy between the two is considered a good measure of body dissatisfaction [6]. Positive scores are indicative of the desire to have a larger body size, while the negative ones are indicative of the desire to be thinner. The only difference between FRS [4] and CDRS [5], is the type of images used, more "comic" those used by Stunkard, Sorensen and Schulsinger and more realistic those used by Thompson and Gray.

Figure or image rating scales may be intended as implicit measures of body dissatisfaction, because they are designed to capture the to-be-measured construct under automaticity conditions [7]. Both scales have the advantages of being quickly administered, showing a strong correlation with the body mass index (BMI) and of being able to be used independently of the culture to which they belong, without the need for either linguistic or cultural adaptation. However, they present a standard limitation: they use schematic figures that are scarcely realistic and, in any case, very tied to a Caucasian ethnicity.

To overcome these limitations, some authors have developed new analogue tools that use photographic support. For example, Swami et al. [8] developed and validated the Photographic Figure Rating Scale (PFRS), an enhanced version of the FRS with the help of photographic images, and therefore, more realistic. Although this instrument has gained in terms of greater realism, at the same time, it has lost in universality, since photographs can hardly be applied in different cultures and ethnic groups.

This study aims to validate a tool for assessing body perception and dissatisfaction in a sample of adult females and males (+ 18). This pictorial tool, the Silhouette Rating Scale (SRS) was created for showing a series of silhouettes (nine for females and nine for males) that guarantees the universality of use thanks to the absence of details related to ethnicity or culture and at the same time, maintaining a right level of realism. Our aims are to test the concurrent, convergent and discriminant validity of the scale, evaluating possible gender differences. To evaluate the concurrent validity we expect: (1) positive correlations between current body size rating and BMI; (2) positive correlations between ideal body size rating and body dissatisfaction score controlling for BMI, (3) significant correlations among the discrepancy between current and ideal body size ratings and two different self-report scales assessing body dissatisfaction. To evaluate the convergent validity we expect: positive correlations between our scale (SRS) and the CDRS [5]. Moreover, in order to evaluate the discriminant validity, we also expect low or no correlations between the SRS measures and other measures of eating disorder-related psychopathology, depression, insomnia and emotion regulation.

## Materials and method

### Participants and procedure

A first sample of 754 young adults (mean age = 26.10 ± 8.50; females N = 536, males *N* = 218) and a second sample of 210 young adults (mean age = 21.19 ± 3.22; females *N* = 167, males *N* = 43) were recruited among the student community of Sapienza University of Rome, using a convenience sampling procedure. All participants voluntary agreed to participate and signed a written informed consent. They were then asked to complete a battery of self-report questionnaires. The study was approved by the Institutional Review Board of the Department of Psychology at Sapienza University of Rome.

### Measures

All participants completed the SRS that presents different images depending on gender. The two scales include nine feminine or masculine silhouettes, varying for body dimensions (width of body parts) and shape: the first one is the thinnest and the ninth is the larger (both scales are available upon request). A professional artist was asked to draw the silhouettes representing adult figures without any ethnical characteristics or facial expression, following the same procedure and modality of another scale developed for children by the same authors [9]. Each scale includes two identical sequences accompanied by different instructions: the first was: “*Please, observe the nine figures and select the one that you perceive as most accurately depicting your current body size*”. While the second one asked: “Please, *observe the nine figures and select the one that that you perceive as most accurately depicting how you would like to be*” (the two scales are included as supplementary material). A score was assigned to each silhouette starting from one (assigned to the thinnest) to nine (assigned to the largest). Based on previous research, the response given to the first set of stimuli was considered a measure of one's current body shape and size (SRS-C); the response given to the second set of stimuli was considered a measure of one’s ideal body shape and size (SRS-I). The discrepancy between the second and the first response (ideal—current body shape and size ratings) was considered a measure of body dissatisfaction (SRS-D). Zero scores indicated no discrepancy between ideal and current body shape and size, possibly indicating no body dissatisfaction, scores above zero reflected the desire to have a larger or fatter body, while scores below zero reflected the desire to have a smaller or thinner body.

All participants also completed the Disordered Eating Questionnaire (DEQ) [10], a 24-item instrument assessing self-reported dysfunctional eating-related behavior patterns and anthropometric measures. Self-reported height and weight were used to calculate Body Mass Index (BMI = weight/height^2^), that has been demonstrated to be a valid and reliable proxy of actual BMI [11]. Moreover, a previous study evidenced that the DEQ produces a valid and reliable global score of disordered eating related behaviors [11]. Items includes both questions regarding disordered eating habits (e.g. during the past three months, how many times it happened: “to limit the quantity of food or calories consumed in order to reduce your weight”; “feeling guilty after eating”; “not to resist the urge of eating a specific food”; “to induce vomit to control your weight” etc.) and questions regarding body dissatisfaction (e.g. during the past three months, how many times it happened: “to feel discomfort in showing your body to others”; “to spend a lot of time thinking about your weight or about the appearance of specific body parts”; “to feel discomfort in seeing your body reflected on a mirror”). In the present study we computed a score from the six items assessing body dissatisfaction related attitudes and behaviors, Cronbach’s alfa being 0.90.

The second sample (*N* = 210) completed also the Contour Drawing Rating Scale [5]; the Body Dissatisfaction Scale of the Eating Disorder Inventory (EDI-II) [12], the Eating Attitude Test (EAT-26) [13], the Beck Depression Inventory (BDI-I) [14] and the Emotion Regulation Questionnaire (ERQ) [15] and the Insomnia Severity Index [16].

The CDRS is a widely used analogic scale developed by Thompson and Gray [5] which presents the same structure of our scale but different images: it is composed by nine figures depicting images of bodies of various sizes and appropriate for gender. Participant are first asked to select which image represents the actual size of their own body and secondly, which image represents the ideal body size.

The Body Dissatisfaction Scale of the EDI-II is a 9-items scale which measures concerns about body shape and dissatisfaction with specific body parts, derived from the Eating Disorder Inventory-II [12], a 91-items questionnaire assessing eating disorders symptoms and related psychological characteristics. Cronbach’s alfa in this study was 0.88.

The EAT-26 [13] is a 26-item scale assessing eating disorders-related symptoms and concerns. In the present study, we used the Italian version validated by Dotti and Lazzari [17]. It consists of 3 subscales: “dieting”, “bulimia and food preoccupation” and “oral control”. The factorial structure of the Italian version, although similar, does not fully correspond to the original one and the reliability of two of the three subscales is low (Cronbach's α of 0.87; 0.70; 0.62, respectively) [17]. For this reason, we followed authors’ suggestion to calculate a total score which showed good reliability in the validation study (α = 0.86). In the present study the Cronbach’s alfa of the total score was 0.883.

The BDI-II [14], in the Italian version validated by Sica and Ghisi [18], is a 21-item scale largely used to assess the presence and severity of depressive symptoms. Cronbach’s α in the English validation study was 0.91 while in the present study Cronbach’s alfa was 0.93.

The ERQ, in the Italian version by Balzarotti et al. [19], is a brief scale measuring the use of two emotion regulation strategies trough two subscales: expressive suppression and cognitive reappraisal. Research indicates that the scale is internally consistent (α reliability coefficients were 0.73 and 0.79 in the validation study [20] and displays strong convergent and discriminant validity [15]. In the present study Cronbach’s alfa were, respectively, 0.72 and 0.86 for the two subscales.

Finally, the ISI [16] in the Italian version of Battagliese and Lombardo [21] was employed to provide a measure of insomnia severity during the preceding two weeks. The brief scale includes items regarding difficulty falling asleep, difficulty staying asleep, problems waking up too early, and worries and satisfaction regarding sleep pattern and daily functioning. Cronbach’s α in the validation study was 0.76 while in this study was 0.83.

### Statistical analyses

#### Analyses on the first sample

For the first sample (*N* = 754) descriptives of the two gender groups were performed, as well as differences between them using Anovas. In addition, participants were divided using BMI categories: underweight (BMI < 18.50), normal weight (BMI = 18.50–24.99) and overweight/obesity (BMI > 25.00), based on World Health Organization guidelines. Differences across BMI categories and genders were computed considering all the measured variables.

Then, to examine the concurrent validity of the current body size and shape ratings, bivariate correlations between BMI and SRS-C were computed separately for gender.

After that, to evaluate the concurrent validity of the measure of the ideal perception of body size and shape, partial correlations between the Body Dissatisfaction Scale of the DEQ and SRS-I were computed separately for gender and controlling for BMI.

Finally, in order to evaluate the concurrent validity of body dissatisfaction score (SRS-D), we explored the bivariate correlations between this measure and the Body Dissatisfaction Scale of the DEQ.

#### Analyses on the second sample

For the second sample (*N* = 210), in order to measure convergent validity of the scale, bivariate correlations with each measure of the scale (SRS-C, SRS-I, SRS-D) and the corresponding scores obtained from the Contour Drawing Rating Scale (CDRS-A, CDRS-I, CDRS-D) were performed. Concurrent validity was then evaluated using bivariate correlations between SRS-A and BMI. In the same way, concurrent validity between SRS-D, CDRS-D and Body Dissatisfaction Scale of the EDI-II was explored. Finally, discriminant validity was tested exploring bivariate correlations between scores obtained from the analogic scale (SRS-C, SRS-I, SRS-D) and scores at EAT-26, BDI-II, ISI, ERQ.

## Results

### First sample

Table 1 shows the descriptives of the first sample across genders and the statistical comparisons of the two groups. Males report older age, higher BMI and lower body dissatisfaction than females.

**Table 1:** Descriptives and differences between females and males

Variables	Males (*N* = 218)	Females (*N* = 536)	*F*_(753)_	*p*	Partial* η*^2^
Age	28.25 ± 10.39	25.22 ± 7.44	20.13	< 0.001	0.026
BMI	23.72 ± 3.39	21.49 ± 3.31	69.34	< 0.001	0.085
Current body size rating (SRS-C)	5.42 ± 1.76	5.89 ± 1.84			
Ideal body size rating (SRS-I)	5.27 ± 1.01	4.50 ± 1.39			
Body dissatisfaction score (SRS-D)	− 0.15 ± 1.50	− 1.39 ± 1.35			
Body dissatisfaction score of DEQ	5.88 ± 6.14	10.94 ± 8.69	61.55	< .001	0.076

Male and female version of the SRS were considered as two separate scales, since the silhouettes representing adult figures were different for male and female. For this reason, no comparison across genders was performed on these measures. Differences between BMI categories

In the male subgroup, eight participants were underweight, 149 normal weight and 60 overweight or obese. In the female subgroup 70 participants were underweight, 407 normal weight and 57 overweight or obese. Comparisons between BMI groups in males were performed only considering normal weight and overweight/obese, due to the small number of underweight males. Mean scores are displayed in Table 2 together with the results divided per gender and BMI categories.

**Table 2:** Differences across BMI categories and genders

Males (*N* = 218)	Underweight (*N* = 8)^a^	Normal weight (*N* = 149)	Overweight/ obese (*N* = 60)	*F*	*p*	Partial * η*^2^
SRS-C	3.75 ± 1.04	4.81 ± 1.44	7.22 ± 1.11	75.65	< 0.001	0.34
SRS-I	5.00 ± 2.00	5.11 ± 0.96	5.68 ± 0.87	7.43	0.001	0.07
SRS-D	1.25 ± 1.98	0.31 ± 1.29	− 1.53 ± 0.85	54.75	< 0.001	0.34
Body dissatisfaction score of DEQ	8.25 ± 7.59	4.96 ± 5.58	7.60 ± 6.61	4.92	0.008	0.04

Females (*N* = 536)	Underweight (*N* = 70)	Normal weight (*N* = 407)	Overweight/ obese (*N* = 57)	*F*	*p*	Partial * η*^2^
SRS-C	3.80 ± 1.50^a^	5.92 ± 1.56^b^	8.19 ± 0.90^c^	136.38	< 0.001	0.41
SRS-I	3.51 ± 1.15^a^	4.47 ± 1.31^b^	5.93 ± 1.05^c^	58.13	< 0.001	0.18
SRS-D	− 0.28 ± 1.41^a^	− 1.45 ± 1.25^b^	− 2.26 ± 1.16^c^	40.75	< 0.001	0.13
Body dissatisfaction score of DEQ	8.36 ± 8.61^a^	10.64 ± 8.17^b^	16.31 ± 10.25^c^	14.98	< 0.001	0.05

^a^Results of Anovas performed in the male group refer only to the comparison between normal weight and overweight/obese

*a, b, c *LSD post hoc comparisons indicating means differing at *p* <0 .001, *SRS-C *current body size evaluation of the Silhouette Rating Scale, *SRS-I *ideal body size evaluation of the Silhouette Rating Scale, *SRS-D *body dissatisfaction as the discrepancy between the ideal and current body size evaluation of the Silhouette Rating Scale, *DEQ* Disordered Eating Questionnaire

### Concurrent validity

#### Current body size rating

Results of the Pearson’s bivariate correlations between BMI and the rating of the current body size/shape (SRS-C) showed that the two variables are significantly correlated both in males (*r* = 0.72, *p* < 0.001) and in females (*r* = 0.70, *p* < 0.001). This pattern of results indicates that the higher the BMI, the larger is the figure selected.

#### Ideal body size rating

Partial correlations between the ideal body size rating (SRS-I) and the Body Dissatisfaction score of the DEQ, controlling for BMI, evidenced a significant negative correlation in females (*r* = −0.21, *p* < 0.001). This result indicates that the thinnest is the ideal body size, the higher is the body dissatisfaction reported. Conversely, this correlation was not significant in males (*r* = 0.02, *p* = 0.753).

#### Body size dissatisfaction

Pearson’s bivariate correlations between the body dissatisfaction score (SRS-D) (i.e. the discrepancy between the ideal and the current body size ratings) and the score on the Body Dissatisfaction computed from the DEQ were significant and negative both in males (*r* = −0.24, *p* < 0.001) and in females (*r* = −0.57, *p* < 0.001). Moreover, bivariate correlation between the body dissatisfaction score (SRS-D) and BMI were significant and negative both in males (*r* =−0.63, *p* < 0.001) and in females (*r* = −0.41, *p* < 0.001).

### Second sample

#### Convergent, concurrent and discriminant validity

Statistical analyses conducted on the second sample (total *N* = 210) consisting of 167 females (age *M* = 20.80 ± 2.42) and 43 males (age *M* = 22.67 ± 5.16) revealed significant positive correlations between the responses at the SRS-C, SRS-I, SRS-D and the responses at the CDRS-C, CDRS-I, CDRS-D in both genders, supporting the convergent validity. Moreover, the correlation between current body size measured by the two scales and BMI (SRS-C and BMI vs CDRS-C and BMI) are comparable, indicating that both scales show good concurrent validity. In addition, both scores of SRS-D and CDRS-D are strongly negatively correlated with the Body Dissatisfaction Scale of the EDI-II. Results are shown in Table 3.

**Table 3:** Pearson’s correlations between the scores of current and ideal body size, and body dissatisfaction computed from the SRS and the CDRS

Females (*N* = 167)	CDRS-C	CDRS-I	CDRS-D	BMI	EDI-BD
SRS-C	0.84†			0.77†	
SRS-I		0.70†			
SRS-D			0.74†		− .65†
BMI	076†				
			− 0.74†		

Males (*N* = 43)	CDRS-C	CDRS-I	CDRS-D	BMI	EDI-BD
SRS-C	0.95†			0.74†	
SRS-I		0.79†			
SRS-D BMI			0.94†		− 0.31*
	0.75†				
EDI-BD			− 0.290		

*SRS-C *current body size evaluation of the Silhouette Rating Scale, *SRS-I *ideal body size evaluation of the Silhouette Rating Scale, *SRS-D *body dissatisfaction as the discrepancy between the ideal and current body size evaluation of the Silhouette Rating Scale, *BMI* body mass index, *EDI-BD *Body Dissatisfaction Scale of Eating Disorder Inventory-II, *CDRS-I *cCurrent body size evaluation of the Contour Drawing Rating Scale, *CDRS-I *ideal body size evaluation of the Contour Drawing Rating Scale, *CDRS-D *body dissatisfaction as the discrepancy between the ideal and current body size evaluation of the Contour Drawing Rating Scale

*p < 0.05, **p < 0.01, †*p* ≤ 0.001

Finally, to support the discriminant validity of our scale, Table 4 presents the Pearson’s bivariate correlation coefficients among the three scales (SRS-C, SRS-I, SRS-D) and the other measures of eating disorder symptoms (EAT-26), depression (BDI-II), insomnia (ISI) and emotion regulation (ERQ expressive suppression, ERQ cognitive reappraisal), separated per gender. No significant correlations were found in males, except for the correlation between SRS-I and EAT-26 (*r* = −0.49, *p* = 0.001), suggesting that thinner ideal body size selection was associated with higher eating disorders symptomatology. Among the female sample, significant correlations were found: (1) a positive correlation between the score of SRS-C and EAT-26 (*r* = 0.15, *p* = 0.049), indicating that higher eating disorders symptoms were associated with larger current body size rating; (2) a negative significant correlation between the score of SRS-D and EAT-26 (*r* = −0.34, *p* < 0.001), indicating that body dissatisfaction, in term of the desire to be thinner, was associated with higher level of eating disorders symptomatology; (3) a marginal negative correlation between SRS-I and EAT-26 (*r* = -0.15, *p* = 0.053); (4) significant negative correlations were found between SRS-D and expressive suppression (*r* = −0.18, *p* = 0.022) and between SRS-D and BDI-II (*r* = −0.20, *p* = 0.010), indicating that this variable was associated also to higher levels of depressive symptomatology and higher use of the expressive suppression.

**Table 4:** Pearson’s correlation coefficients among scores of the pictorial figure rating scale and the other measures of eating disorders symptoms, depression, insomnia and emotion regulation

Females (*N* = 167)	EAT-26	BDI-II	ISI	ERQ (expressive suppression)	ERQ (cognitive reappraisal)
SRS-C	0.15*	0.14	− 0.01	0.11	− 0.06
SRS-I	− 0.15	− 0.02	− 0.10	− 0.05	− 0.05
SRS-D	− 0.34†	− 0.20**	0.08	− 0.18*	003

Males (*N* = 43)	EAT-26	BDI-II	ISI	ERQ (expressive suppression)	ERQ (cognitive reappraisal
SRS-C	− 0.19	− 0.04	− 0.06	− 0.12	− 0.11
SRS-I	− 0.49†	− 0.19	− 0.05	− 0.26	0.08
SRS-D	− 0.08	− 0.09	0.11	− 0.02	0.29

*SRS-C *current body size evaluation of the Silhouette Rating Scale, *SRS-I *ideal body size evaluation of the Silhouette Rating Scale; *SRS-D *body dissatisfaction as the discrepancy between the ideal and current body size evaluation of the Silhouette Rating Scale, *EAT-26* Eating Attitude Test-26, *BDI-II* Beck Depression Inventory-II, *ISI* Insomnia Severity Index, *ERQ *Emotion Regulation Questionnaire

*p < 0.05, **p < 0.01, *†p* ≤ 0.001

Finally, *r* values in support to the convergent, concurrent and discriminant validity were also compared using the *z* test (computed according to Eid, Gollwitzer and Schmidt [22]) to verify significance of the differences between the correlation coefficients. Therefore, comparing correlations indicating convergent and discriminant validity, their standardized differences are between Z = 11.20 and Z = 7.36 (all ps < 0.001) for the female sample, while in the male sample Z values ranged between 16.32 and 9.19 (all ps < 0.001). Comparing correlations indicating concurrent and discriminant validity values, Z ranged between 9.67 and 7.74 for females and between 9.45 and 8.37 for males (al ps < 0.001). All differences were in the expected direction thus supporting convergent, concurrent and discriminant validity of the scale.

## Discussion

The present study aimed at validating a new Pictorial Rating Scale, including nine females and nine males silhouettes and evaluating its concurrent, convergent and discriminant validity across a first sample of 754 and a second sample of 210 young adults.

Considering results about the current body size, it was correlated with BMI both in males and in females showing a strong concurrent validity, with a large effect in both samples [23, 24]. In addition, the correlation coefficient between current body size measured by our scale and BMI in the second sample was similar with that one between current body size measured by Thompson’s scale and BMI, with a large effect. This supports the concurrent validity of both scales, indicating that the current body size scale of the SRS may be a valid new and alternative tool to assess current body size.

Furthermore, the ideal body size evaluation showed a significant negative correlation with the Body Dissatisfaction score of the Disordered Eating Questionnaire, with a small effect in females but not in males. This finding could be related to the adhesion to the thin ideal, which characterizes the society as demonstrated by Rochelle and Hu [25]. They found that exposure to thin-ideal media produced an increase in the drive for thinness, body dissatisfaction and problematic eating attitudes. In our data, this correlation is present independently from the BMI, suggesting that consistently with previous findings, even normal weight persons would be dissatisfied with their own body and would desire to be thinner [26], especially women, regardless of shape or size, are dissatisfied with their bodies [27]. However, the absence of significant correlations between ideal body size and body dissatisfaction in males may probably reflect that media and masculine norms drive for muscularity in shaping men’s body [28, 29], contrarily to the females’ thin-ideal, or that other underline mechanisms are involved.

The concurrent validity is also supported by the significant negative correlations: (a) between the body dissatisfaction score and the body dissatisfaction score of the Disordered Eating Questionnaire in both genders in the first sample; (b) between the body dissatisfaction score and the score of Body Dissatisfaction Scale of Eating Disorder Inventory-II in both genders in the second sample. Results from these correlations are similar to those detected using Thompson’s scale, again supporting that both scales are valid and reliable measures of implicit body dissatisfaction. The amplitude of the coefficients detected in females (−0.65 and −0.75), compared to those found in males (−0.31 and −0.29), suggests that this scale is likely more fat-sensitive than shape-sensitive, and supports the well-known difference in the beauty ideals and body dissatisfaction among females and males.

Also, convergent validity was largely supported by the positive significant correlations between the three measures of our scale and the three measures of the Thompson’s scale. Large effects were found (ranging from 0.70 to 0.95) between the six ratings analyzed. This new tool, compared to Thompson’s scale, has the advantages to not present details related to ethnicity or culture guaranteeing the universality of use, and maintaining at the same time a good level of realism.

In addition, the correlations computed in the second sample between the three responses of the SRS and other measures of eating disorders, depression, insomnia and emotion regulation indicated a good discriminant validity, though some of the variables measured seem to be significantly correlated (low to moderated effect according to Cohen’s guidelines [24]). Specifically, in the female group current body size and body dissatisfaction, as well as ideal body size in male group, were associated with eating disorder symptomatology. The relationship between body dissatisfaction, thin ideal, current boy size and eating disorders is well known, and they are considered risk factors predicting eating disorder symptom onset [30]. In the male group, only thinner ideal body size was associated with eating disorders (with moderate effect), probably because the processes underlying the etiology of specific eating disorders may be different for males and females [31]. Moreover, it has been suggested that for males the ideal body is synonymous of bigger and more muscular body. Thus, they could desire to increase their muscularity for feeling more attractive and dominant [32]. Finally, the link found between body dissatisfaction and depression has been documented, especially in females [25, 33]. The link between body dissatisfaction and expressive suppression, which may be considered a maladaptive strategy connected with negative health outcomes [34], is still poorly studied, although few indirect evidence may be derived from a study demonstrating positive effects of adaptive emotion regulation strategies on body dissatisfaction [35].

This study presents several limitations such as the use of self-report instruments and the lack of anthropometric measures, i.e. for the BMI calculation. Moreover, the scarce number of males in the second sample may not offer a wide picture of the general population, although part of the results extends the findings obtained in the first sample. Also, the lack of age and ethnic categories limits the extension of our results to the general population. Finally, the use of the scale only within a non-clinical population does not allowed to extend our results to the clinical population, especially among individuals with eating disorders. Future studies addressing these limitations will be useful to extend and confirm its validity and reliability, likewise, measuring its test–retest validity.

## What is already known on this subject?

So far, numerous instruments assessing body image have been produced. Most of the analogic scales available present a limitation: they use scarcely realistic figures tied to Caucasian ethnicity.

## What does this study add?

The Silhouette Rating Scale is a reliable and valid tool for assessing current and ideal body size compared to other available scales. It guarantees the universality of use and a good level of realism.

## Supplementary Information

Below is the link to the electronic supplementary material.

Supplementary file1 (PDF 274 KB)

## References

[1] Cash TF, Smolak L (2011). Body image: a handbook of science, practice, and prevention.

[2] Kling J, Kwakkenbos L, Diedrichs PC (2019). Systematic review of body image measures. Body Image.

[3] Shroff H, Calogero R, Thompson J (2009) Assessment of body image. In: Handbook of assessment methods for eating behaviors and weight-related problems measures, theory, and research, 2nd edn, pp 115–136

[4] Stunkard AJ, Sørensen T, Schulsinger F (1983). Use of the Danish Adoption Register for the study of obesity and thinness. Res Publ Assoc Res Nerv Ment Dis.

[5] Thompson MA, Gray JJ (1995). Development and validation of a new body-image assessment scale. J Pers Assess.

[6] Gleaves DH, Lowe MR, Green BA (2000). Do anorexia and bulimia nervosa occur on a continuum? A taxometric analysis. Behav Ther.

[7] De Houwer J, Teige-Mocigemba S, Spruyt A, Moors A (2009). Implicit measures: a normative analysis and review. Psychol Bull.

[8] Swami V, Hadji-Michael M, Furnham A (2008). Personality and individual difference correlates of positive body image. Body Image.

[9] Lombardo C, Battagliese G, Pezzuti L, Lucidi F (2014). Validity of a figure rating scale assessing body size perception in school-age children. Eat Weight Disord.

[10] Lombardo C, Russo PM, Lucidi F (2004). Internal consistency, convergent validity and reliability of a brief questionnaire on disordered eating (DEQ). Eat Weight Disord.

[11] Lombardo C, Cuzzolaro M, Vetrone G (2011). Concurrent validity of the Disordered Eating Questionnaire (DEQ) with the Eating Disorder Examination (EDE) clinical interview in clinical and non-clinical samples. Eat Weight Disord.

[12] Garner DM (1991) Eating disorder inventory-2: professional manual. Psychological Assessment Resources, Odessa, Florida, P.O. Box 998, Odessa 33556

[13] Garner DM, Olmsted MP, Bohr Y, Garfinkel PE (1982). The Eating Attitudes Test: psychometric features and clinical correlates. Psychol Med.

[14] Beck AT, Steer RA, Brown GK (1996). BDI-II, Beck depression inventory: manual San Antonio.

[15] Gross JJ, John OP (2003). Individual differences in two emotion regulation processes: implications for affect, relationships, and well-being. J Pers Soc Psychol.

[16] Bastien CH, Vallières A, Morin CM (2001). Validation of the Insomnia Severity Index as an outcome measure for insomnia research. Sleep Med.

[17] Dotti A, Lazzari R (1998). Validation and reliability of the Italian EAT-26. Eat Weight Disord.

[18] Sica C, Ghisi M (2007). The Italian versions of the Beck Anxiety Inventory and the Beck Depression Inventory-II: psychometric properties and discriminant power. Leading-edge psychological tests and testing research.

[19] Balzarotti S, John OP, Gross JJ (2010). An Italian adaptation of the emotion regulation questionnaire. Eur J Psychol Assess.

[20] Melka SE, Lancaster SL, Bryant AR, Rodriguez BF (2011). Confirmatory factor and measurement invariance analyses of the emotion regulation questionnaire. J Clin Psychol.

[21] Battagliese G, Lombardo C (2012) Insomnia severity index. In: Coradeschi D, Devoto A (eds) Insonnia. Strumenti di valutazione psicologica. In Quaderni di Psicoterapia Cognitiva e Comporta- mentale. Erickson Edizioni, Trento, pp 23–32

[22] Eid M, Gollwitzer M, Schmitt M (2011). Statistik und Forschun-gsmethoden [Statistics and Research Methods].

[23] Cohen J (1988) Statistical power analysis for the behavioral sciences, 2nd edn. L. Erlbaum Associates, Hillsdale

[24] Cohen J (1992). A power primer. Psychol Bull.

[25] Rochelle TL, Hu WY (2017). Media influence on drive for thinness, body satisfaction, and eating attitudes among young women in Hong Kong and China. Psychol Health Med.

[26] Hagman J, Gardner RM, Brown DL (2015). Body size overestimation and its association with body mass index, body dissatisfaction, and drive for thinness in anorexia nervosa. Eat Weight Disord.

[27] Tiggemann M (2011) Sociocultural perspectives on human appearance and body image. In: Cash TF, Smolak L, Cash TF, Smolak L (eds) Body image. A handbook of science, practice, and prevention, 2nd edn. Guilford Press, New York

[28] De Jesus AY, Ricciardelli LA, Frisén A (2015). Media internalization and conformity to traditional masculine norms in relation to body image concerns among men. Eat Behav.

[29] Ralph-Nearman C, Filik R (2018). New body scales reveal body dissatisfaction, thin-ideal, and muscularity-ideal in males. Am J Mens Health.

[30] Stice E, Gau JM, Rohde P, Shaw H (2017). Risk factors that predict future onset of each DSM-5 eating disorder: predictive specificity in high-risk adolescent females. J Abnorm Psychol.

[31] MacNeill LP, Best LA, Davis LL (2017). The role of personality in body image dissatisfaction and disordered eating: discrepancies between men and women. J Eat Disord.

[32] Frederick DA, Buchanan GM, Sadehgi-Azar L (2007). Desiring the muscular ideal: men's body satisfaction in the United States, Ukraine, and Ghana. Psychol Men Masc.

[33] Wiederman MW, Pryor TL (2000). Body dissatisfaction, bulimia, and depression among women: the mediating role of drive for thinness. IntJ Eat Disord.

[34] Hu T, Zhang D, Wang J (2014). Relation between emotion regulation and mental health: a meta-analysis review. Psychol Rep.

[35] Prefit AB, Cândea D-M, Szentagotai-Tătar A (2020). Effects of acceptance and reappraisal on body dissatisfaction: an experimental comparison of two adaptive emotion regulation strategies. Eat Weight Disord.

