# Supplementary material for: Psychometric properties of a Silhouette Rating Scale assessing current and ideal body size and body dissatisfaction in adults
Source: Eat Weight Disord. 2021 Jul 8;27(3):1089–97. doi: 10.1007/s40519-021-01258-6 (PMC8964547; doi:10.1007/s40519-021-01258-6)
Supplement: Supplementary file 1 — Supplementary file1 (PDF 274 KB) [file 40519_2021_1258_MOESM1_ESM.pdf]

Silhouette Rating Scale (SRS, Lombardo, Cerolini, Esposito & Lucidi)

ITA: 1) Osserva le figure che seguono e indica con una crocetta quale figura rappresenta **COME SEI**

ENG: 1) Please, observe the nine figures and select the one that you perceive as most accurately depicting your **current body size**

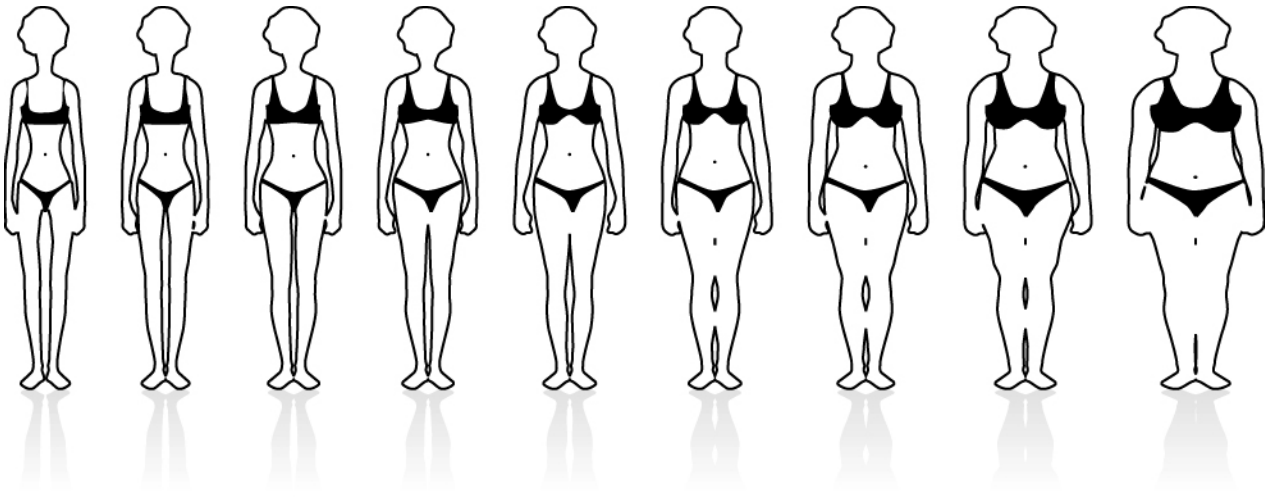

1 ☐ 2 ☐ 3 ☐ 4 ☐ 5 ☐ 6 ☐ 7 ☐ 8 ☐ 9 ☐

ITA: 2) Osserva le figure che seguono e indica con una crocetta quale figura rappresenta **COME VORRESTI ESSERE**

ENG: 2) Please, observe the nine figures and select the one that that you perceive as most accurately depicting how **you would like to be**

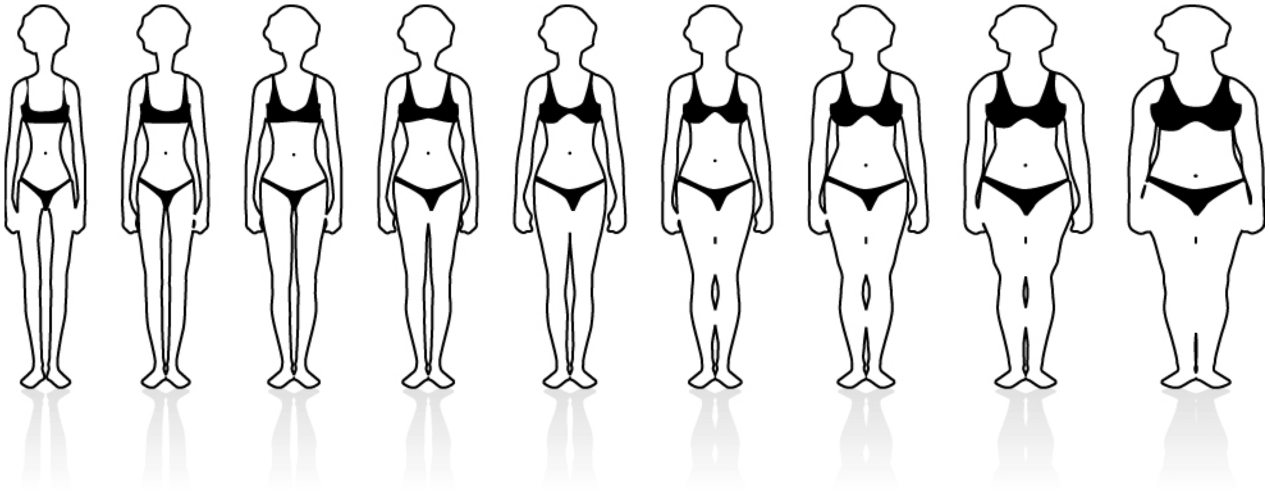

1 ☐ 2 ☐ 3 ☐ 4 ☐ 5 ☐ 6 ☐ 7 ☐ 8 ☐ 9 ☐

ITA: 1) Osserva le figure che seguono e indica con una crocetta quale figura rappresenta **COME SEI**

ENG: 1) Please, observe the nine figures and select the one that you perceive as most accurately depicting your **current body size**

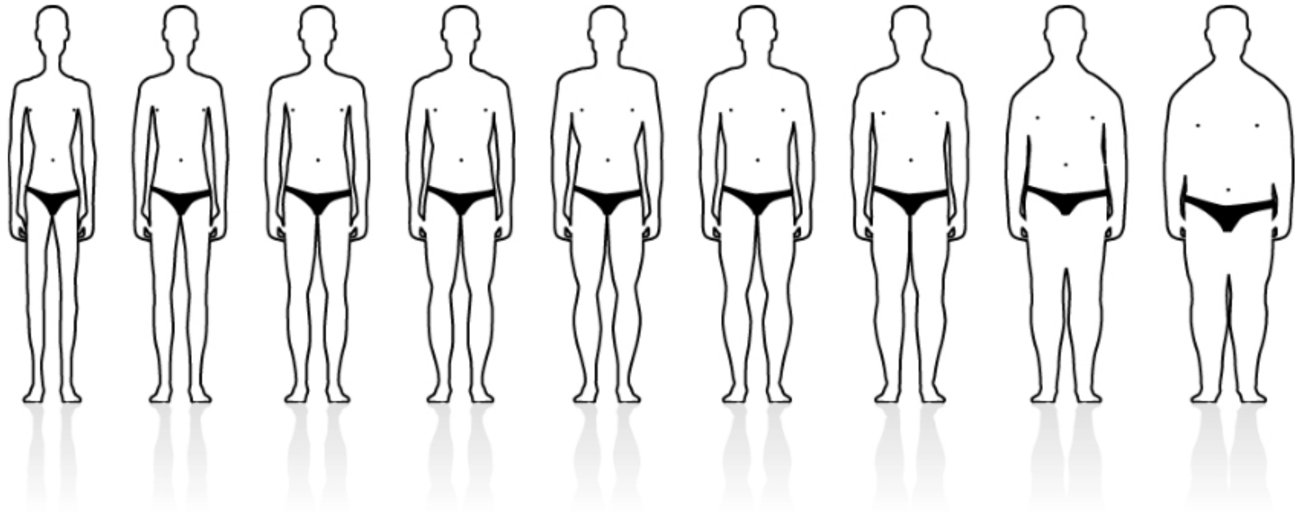

1 ☐ 2 ☐ 3 ☐ 4 ☐ 5 ☐ 6 ☐ 7 ☐ 8 ☐ 9 ☐

ITA: 2) Osserva le figure che seguono e indica con una crocetta quale figura rappresenta **COME VORRESTI ESSERE**

ENG: 2) Please, observe the nine figures and select the one that that you perceive as most accurately depicting how **you would like to be**

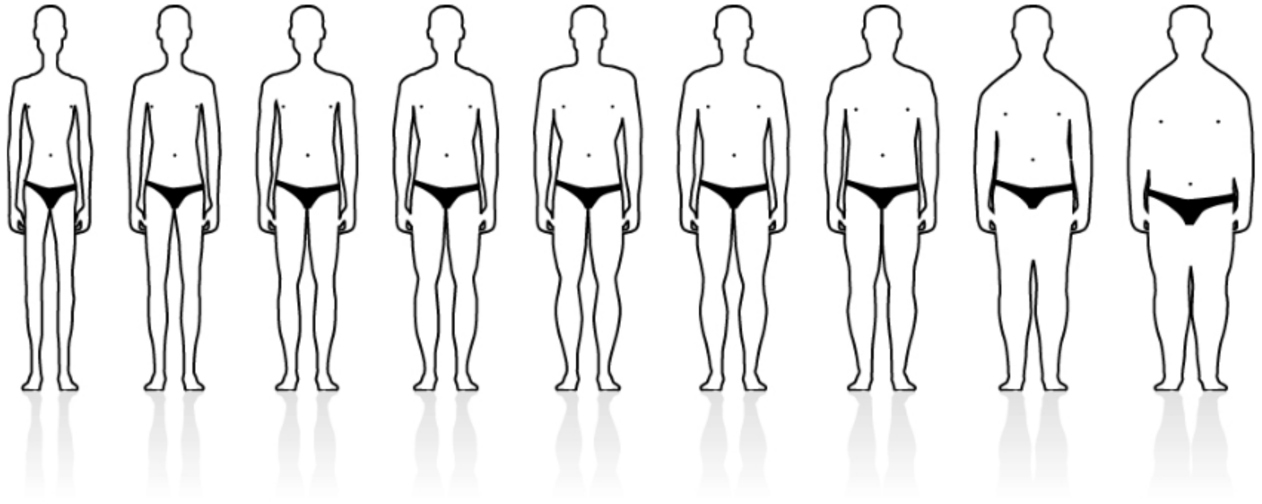

1 ☐ 2 ☐ 3 ☐ 4 ☐ 5 ☐ 6 ☐ 7 ☐ 8 ☐ 9 ☐
